# Presidential Symposium: From Disruption to Transformation: Challenging and Changing the New Normal

**DOI:** 10.1093/geroni/igab046.969

**Published:** 2021-12-17

**Authors:** Deborah Waldrop, Philip Rozario, Emily Greenfield

## Abstract

While the refrain “We’re all in this together” is meant to describe a sense of universality of our exposure and adaption to the Covid-19 pandemic life, the deeply rooted racial and economic injustices and ongoing health crises continue to expose the inequities experienced by many older adults. In this symposium, we focus on existing disparities and possibilities for transformation. The first paper discusses systemic racism as a structural driver of practices and policies that influence poverty, poor housing and neighborhood conditions, worse health profiles, relationship loss and social isolation among older Black adults. The second paper illuminates the importance of health equity and collaboration between aging and healthcare systems to improve the well-being outcomes and address disparities of older adults from racial-ly/ethnically diverse backgrounds. The third paper illustrates how the privatization of Medicare has created bureaucratic complexities that increase cost and burdens for beneficiaries. The fourth paper presents the ways that the pandemic has exposed the challenges of a nonexistent Long Term Services and Support system; specifically, in refocusing our attention on the working conditions of in-home and residential workers, such as poor compensation, and high turnover and mounting demands on families. The fifth paper addresses the importance of collaboration between nursing homes and assisted living communities with governmental emergency operations in times of disasters and public health crises. Each paper addresses pressing issues that have created the “new normal” for older adults; together the presenters explore the disruptions and offer solutions for renewed transformation.

